# In-cell western assay as a high-throughput approach for *Chlamydia trachomatis* quantification and susceptibility testing to antimicrobials

**DOI:** 10.1371/journal.pone.0251075

**Published:** 2021-05-11

**Authors:** Simone Filardo, Marisa Di Pietro, Patrizio Pasqualetti, Martina Manera, Fabiana Diaco, Rosa Sessa

**Affiliations:** 1 Department of Public Health and Infectious Diseases, Section of Microbiology, University of Rome “Sapienza”, Rome, Italy; 2 Department of Public Health and Infectious Diseases, Section of Health Statistics and Biometry, University of Rome “Sapienza”, Rome, Italy; University of Virginia School of Medicine, UNITED STATES

## Abstract

*Chlamydia trachomatis*, the leading cause of bacterial sexually transmitted diseases in developed countries, with around 127 million new cases per year, is mainly responsible for urethritis and cervicitis in women, and urethritis and epididymitis in men. Most *C*. *trachomatis* infections remain asymptomatic (>50%) and, hence, untreated, leading to severe reproductive complications in both women and men, like infertility. Therefore, the detection of *C*. *trachomatis* as well as the antimicrobial susceptibility testing becomes a priority, and, along the years, several methods have been recommended, like cell culture and direct immunofluorescence (DFA) on cell cultures. Herein, we described the application of In-Cell Western assay (ICW) via Odyssey CLx as a fast, more accessible, and high-throughput platform for the quantification of *C*. *trachomatis* and the screening of anti-chlamydial drugs. As a first step, we set up a standard curve by infecting cell monolayers with 2-fold serial dilutions of *C*. *trachomatis* Elementary Body (EB) suspension. Then, different unknown *C*. *trachomatis* EB suspensions were quantified and the chlamydial susceptibility testing to erythromycin was performed, using the DFA as comparison. Our results showed a very high concordance between these two assays, as evidenced by the enumeration of chlamydial IFUs as well as the determination of erythromycin Minimum Inhibitory Concentration (MIC). In conclusion, the ICW assay may be a promising candidate as an accurate and accessible methodology for *C*. *trachomatis* antimicrobial susceptibility testing.

## Introduction

*Chlamydia trachomatis* is the leading infectious cause of blindness among world’s poorest people, and, in developed countries, it is one of the major bacterial sexually transmitted pathogens with approximately 127 million new infections per year [1, 2]. In women, *C*. *trachomatis* manifests as cervicitis and salpingitis while, in men, it is mainly responsible for urethritis [3–5]. Furthermore, it can be transmitted to infants following the direct contact with infective cervical secretions during delivery, resulting in neonatal conjunctivitis and pneumonitis [5].

A major concern with *C*. *trachomatis* is that most urogenital infections are asymptomatic (>50%) and, hence, undetected, and untreated, leading to long-term complications including pelvic inflammatory disease, ectopic pregnancy, and infertility in women as well as epididymitis and proctitis in men [5, 6].

*C*. *trachomatis*, an obligate intracellular bacterium, has an intriguing and unique biphasic developmental cycle alternating between the extracellular, infectious elementary body (EB) and the intracellular, noninfectious, reticulate body (RB) [3, 7, 8]. The developmental cycle begins when EBs attach and enter the host cell by endocytosis. Once inside the host cell, EBs are internalized and confined to a vacuole termed inclusion, through a process requiring the secretion of several proteins including Type-III secretion system effector proteins [7, 9]. Within the inclusion, EBs then differentiate to RBs, which replicate by binary fission within 24 hours post-infection and, as the inclusion expands, RBs begin to transition back to EBs in an asynchronous process [7, 9]. At the end of the developmental cycle, the inclusion occupies most of the host cell’s cytoplasm and, after approximately 48 hours, the EBs are finally released from the host cell mainly by lysis [7, 10].

Given the impact of asymptomatic chlamydial infections on the development of long-term complications as well as the absence of effective vaccines, the detection of *C*. *trachomatis* as well as antimicrobial susceptibility testing becomes a priority, since, in the last decade, several clinical treatment failures to first-line antibiotics have been reported [11, 12].

During the years, several methods have been used for assaying *C*. *trachomatis* genital infections, such as cell culture and direct immunofluorescence assay (DFA), as well as real-time quantitative polymerase chain reaction (PCR) [13–15]. The latter is currently recommended for its high sensitivity (>90%), specificity (≥99%) and short turnaround times [13]. However, one of the main disadvantages of nucleic acid amplification tests is that the target DNA is amplified without discriminating between DNA originating from viable or non-viable *C*. *trachomatis* [13, 14]. Importantly, PCR assay is not utilized, to date, for antimicrobial susceptibility testing [16], although its potential application in this field appears promising [17, 18].

Cell culture has been considered, for a long time, the “gold standard” for its high specificity (100%) as well as the ability of isolating Chlamydial clinical strains, essential for the detection of *C*. *trachomatis* antimicrobial resistance [19]. However, the peculiar intracellular growth of *C*. *trachomatis*, characterized by a long incubation time, and the visualization of chlamydial inclusions across cell monolayers via fluorescence microscopy, have limited its routine use in clinical laboratories [20]. Indeed, the enumeration of chlamydial inclusions is time-consuming, especially when the analysis of multiple samples is performed, as in antimicrobial susceptibility testing. Further issue of microscopic counting is the extensive operator’s technical skill required, especially when cell monolayers are infected with high load of *C*. *trachomatis* and, hence, a defined and randomized number of microscopic fields must be selected [21]. Therefore, the microscopic counting of chlamydial inclusions is surely influenced by investigator’s bias, further compromising the reliability of findings.

To overcome such issues, a novel and fast approach, that utilizes near-infrared laser-based scanning, namely the In-Cell Western assay (ICW), has been proposed for viruses and intracellular bacteria, including *C*. *trachomatis* [22]. The ICW assay is a cell-based technique for intracellular protein detection and has been largely exploited for the quantitative analysis of cellular signaling pathways due to its high accuracy and reproducibility [23].

Herein, we described the application of ICW assay as a high-throughput platform for the quantification of *C*. *trachomatis*, fundamental for screening anti-chlamydial drugs. On this regard, the feasibility of ICW assay for the evaluation of chlamydial susceptibility to erythromycin has been investigated.

## Materials & methods

### Cell line and culture conditions

McCoy cell line (ATCC^®^ CRL-1696^™^, US) was cultured in Dulbecco’s Modified Eagle Medium (DMEM, Corning^™^, US) supplemented with 10% (v/v) foetal calf serum (FCS) (complete medium), at 37°C in humidified atmosphere with 5% CO_2_.

### *C*. *trachomatis* propagation and titration

*C*. *trachomatis* serovar D UW3 (ATCC^©^ VR-885^™^, US) was propagated in McCoy cells as previously described [24]. Briefly, confluent cell monolayers grown in 25 cm^2^ flasks (6x10^6^ cells/well), were infected with chlamydial EBs by centrifugation at 754 x g for 30 min and harvested by scraping after 36 hours of incubation. The suspension containing Chlamydial EBs was, then, added to equal volumes of 4X Sucrose Phosphate (4SP) buffer and stored at −80°C.

For *C*. *trachomatis* titration, confluent cell monolayers, grown on coverslips in 24-well plates (1x10^5^ cells/well), were infected with 10-fold serial dilutions of *C*. *trachomatis* EB suspension by centrifugation at 754xg for 30 min, then washed 3x with PBS and added with complete medium. After 36-hours of incubation at 37°C and 5% CO_2_, infected cell monolayers were washed 3x with PBS and, then, fixed in 96% ice cold methanol for 10 min at -20°C. Chlamydial inclusions were stained by using DFA via a fluorescein isothiocyanate (FITC)-conjugated anti-Chlamydia lipopolysaccharide (LPS) antibody kit (Oxoid, US), following the manufacturer’s instructions. Inclusions were visualized and counted by using a Leica DM5000B fluorescence microscope (Leica, US) at 400× magnification.

### *C*. *trachomatis* quantification via In-cell Western assay

In order to quantify *C*. *trachomatis* by ICW assay we used species-specific chlamydial major outer membrane protein (MOMP) infrared-immunodetection to visualise chlamydial inclusions in cell monolayers. First, we set-up a standard curve by infecting, above described, confluent cell monolayers, grown on two different 96-well cell-culture microplates (1x10^4^ cells/well) (standard polystyrene tissue culture treated gamma-sterilised microplates, Orange Scientific, US and optically clear flat well bottom polystyrene tissue culture treated microplates, Corning^®^, US), with two-fold serial dilutions of *C*. *trachomatis* EB (stock concentration of 4.37x10^7^ EB/mL), from MOI from 1 to 1/2^9^. Then, three unknown chlamydial cell suspensions were diluted 4 times at a ratio of 1:10, 1:100, 1:500 and 1:1000, and used to infect cell monolayers as above described. After 36 hours post infection, infected cell monolayers were washed 3x with PBS and then, fixed in 4% paraformaldehyde (PFA) for 10 min at room temperature and investigated by ICW assay.

At the same time, confluent cell monolayers, grown on coverslips in 24-well cell culture microplates (1x10^5^ cells/well) were infected under the same experimental conditions above described. After 36 hours post infection, infected cell monolayers were washed 3x with PBS and then, fixed with ice cold 96% methanol and analysed by DFA assay.

### Anti-chlamydial drug screening via In-cell Western assay

Confluent cell monolayers grown on either standard microplates or optically clear bottom microplates (1x10^4^ cells/well), were infected with *C*. *trachomatis* at a MOI of 1.0, as above described. Then, infected cells were treated with two-fold serial dilutions of erythromycin (Sigma-Aldrich, US) (from 1 to 1/2^9^) and incubated at 37°C in humidified atmosphere with 5% CO_2_. After 36 hours post infection, treated and untreated cell monolayers were washed 3x with PBS and, then, fixed in 4% PFA and analysed by ICW assay.

At the same time, confluent cell monolayers grown on coverslips in 24-well cell culture microplates (1x10^5^ cells/well), were infected and treated with erythromycin as above described. After 36 hours post infection, treated and untreated cell monolayers were washed 3x with PBS and, then, fixed with ice cold 96% methanol and analysed by DFA assay.

The Minimum Inhibitory Concentration (MIC) was determined by DFA assay and it was defined as the concentration of antibiotic that was one twofold dilution higher than the transition point (MIC_TP_) as described by Suchland et al., 2003 [19].

### Odyssey CLx in-cell western assay

Cell monolayers were stained with a primary mouse monoclonal antibody against species-specific MOMP (Mab6ciii, The Chlamydia Biobank, UK, Cat. No. #CT602) (1:1000 dilution) combined with a secondary goat anti-mouse infrared (IR) Dye 680RD antibody (Licor Biosciences, US) (1:2000 dilution), as previously described [22]. Briefly, following permeabilization with 0.1% triton X-100 in PBS for 8 min at Room Temperature (RT), cell monolayers were incubated with Odyssey Blocking Buffer for 30 min at RT. Then, cell monolayers were incubated with the primary antibody, diluted in Odyssey Blocking Buffer, for 1 hour at RT and washed 3x with PBS containing 0.1% Tween-20. The IR conjugated secondary antibody was then added and cell monolayers were incubated for 1 hour at RT, followed by three final washes with PBS containing 0.1% Tween-20. Multiwell microplates were then analysed on a laser scanner Odyssey CLx near-infrared imaging system (Licor Biosciences, US) at IR 700nm. The Odyssey system was set at 21μm resolution, high scan quality and auto-intensity mode, and images as well as Absolute Units (A.U.) values from each well were acquired using the Licor Image Studio Software (version 3.1). Recorded A.U. values were then exported into Excel (Microsoft, US, version 2010) and uninfected cell monolayers were used for subtracting unspecific and/or background fluorescent signals.

### Direct immunofluorescence assay

Cell monolayers were stained with FITC-conjugated anti-Chlamydia LPS antibody (Oxoid, US), following the manufacturer’s instructions. Briefly, infected McCoy cell monolayers, grown on coverslip in 24-well cell culture plates and fixed with ice cold 96% methanol, were layered with 35μL of FITC-conjugated mouse monoclonal antibody anti-Chlamydia LPS. After 30 min incubation at 37°C, coverslips were washed 3x with PBS, dried at RT and applied on microscope slides (Thermofisher, US). The number of *C*. *trachomatis* IFU/well was determined by counting all microscopic fields using a fluorescence Leica DM5000B microscope (Leica) at 400× magnification [25].

### Statistical analysis

All data are reported as mean ± standard deviation (SD) of at least four replicates from three independent experiments. For each analysis, data distribution was previously assessed and, since usually the clear departures form gaussianity (also test by means of Shapiro-Wilks procedure) were due to right skewness, log-transformation was applied. The correlation and the relationship between absorbance values and fixed concentration (standards) of *C*. *trachomatis* were estimated according to a linear regression model, after verifying that nonsignificant improvement was provided by higher-order models.

To assess differences between means, a General Linear Model was applied, specifying each time the dependent variable as well as the sources of variation (between-measures factors). When appropriate, the interaction between factors was reported and interpreted. Whenever a significant factor was found, Bonferroni procedure was applied to pairwise comparisons to control alpha-inflation.

The threshold of statistically significance was set at 0.05.

## Results

### Set-up and optimization of the ICW assay

Preliminary experiments were performed to determine the appropriate primary antibody as well as the optimal concentrations for the primary and the secondary antibodies, yielding the best signal to noise ratio. McCoy cell monolayers were infected with *C*. *trachomatis* at an MOI of 1 and incubated for 36 hours. Hence, infected cell monolayers were fixed, permeabilized and stained with three different 2-fold serial dilutions (1:500, 1:1000, 1:2000) of the primary antibody anti-*C*. *trachomatis* LPS or anti-*C*. *trachomatis* MOMP, routinely used in the IFU counting, as well as of the secondary antibody anti-mouse (IRDye 680RD). Background signals were obtained from uninfected cells, treated as previously described. Results showed that the combination of the primary antibody against *C*. *trachomatis* MOMP at 1: 1000 dilution factor, with the secondary antibody at 1:2000 dilution factor, provided the highest signal to noise ratio (3.54 as compared to a ratio of <3 in all the other combinations, Table 1) and, therefore, was chosen for further experiments.

**Table 1:** Optimization of the concentrations for the primary and secondary antibodies.

Primary antibody to *C*. *trachomatis* MOMP dilution factors	Secondary antibody IRDye 680RD dilution factors								
	1:500			1:1000			1:2000		
	Background	Signal	Ratio	Background	Signal	Ratio	Background	Signal	Ratio
	(A.U.)	(A.U.)		(A.U.)	(A.U.)		(A.U.)	(A.U.)	
**1:500**	6.4x107 ± 4x106	1.1x108 ± 4x107	1.75	2.2x107 ± 5x106	4.0x107 ± 6x106	1.81	9.5x106 ± 5x105	2.8x107 ± 4x106	2.98
**1:1000**	4.0x107 ± 2x106	6.6x107 ± 9x106	1.64	1.8x107 ± 7x105	2.7x107 ± 5x106	1.51	4.8x106 ± 9x105	1.7x107 ± 5x106	3.54
**1:2000**	2.8x107 ± 2x106	4.6x107 ± 3x107	1.62	8.8x106 ± 7x105	9.1x106 ± 1x107	1.04	4.6x106 ± 8x105	1.2x107 ± 2x106	2.69

Data are expressed as means ± Standard Deviations (SD); MOMP, Major Outer Membrane Protein; A.U., Absolute Units.

Other experiments were performed to determine the time point after infection. McCoy cell monolayers were infected with *C*. *trachomatis* at an MOI of 1 and incubated for 2, 24, 36 or 48 hours. Hence, infected cell monolayers were fixed and processed for ICW assay. Results showed the highest absorbance values at 36 hours (3.76x10^6^ ± 2.24x10^5^ A.U.) as compared to values found at 2 hours (2.1x10^4^ ± 4.03x10^3^), 24 hours (3.06x10^6^ ± 9.8x10^4^ A.U.) and 48 hours (3.08x10^6^ ± 7.95x10^4^ A.U.), representing immature inclusions at 24 hours post infection and cell lysis at 48 hours post infection. At 2 hours post infection, the absorbance values (2.1x10^4^ ± 4.03x10^3^) were similar to those of uninfected cells (2.2x10^4^ ± 9.7x10^2^).

Lastly, further experiments were carried out to determine the appropriate MOI to be used in anti-chlamydial drug screening. In particular, McCoy cell monolayers were infected with *C*. *trachomatis* at MOIs of 0.5, 1.0, 2.0, 5.0 and 10.0 and incubated for 36 hours. Hence, infected cell monolayers were fixed and processed for ICW assay. As reported in Table 2, the results showed that signals were maximum when high MOIs were used. Indeed, absorbance values observed at MOI of 2.0, 5.0 and 10.0 were 1.54x10^8^ ± 3.14x10^6^ A.U., 1.60x10^8^ ± 6.03x10^6^ A.U., 1.53x10^8^ ± 2.74x10^6^ A.U., respectively, showing the inability of the ICW assay to discriminate these different MOI. Therefore, to evaluate the applicability of ICW for *C*. *trachomatis* antimicrobial susceptibility testing, we used the MOI of 1.0 since it possessed the higher signal to noise ratio as compared to MOI of 0.5 (1.47 and 1.04, respectively).

**Table 2:** Determination of the appropriate *C*. *trachomatis* MOI to be used in anti-chlamydial drug screening.

*C*. *trachomatis* MOI	Signal (A.U.)	Signal to noise ratio
**0.5**	7.8x107 ± 1.2x107	1.04
**1**	1.1x108 ± 9.5x106	1.47
**2**	1.5x108 ± 3.1x106	2.06
**5**	1.6x108 ± 6.0x106	2.14
**10**	1.5x108 ± 2.7x106	2.05
**No *Chlamydia***	7.5x107 ± 3.0x106	

MOI, Multiplicity of Infection; A.U., Absolute Units.

Lastly, in order to investigate the stability of the IRdye 680RD, we scanned the same microplates after 1 month of -20°C storage, showing no statistically significant variation of absorbance values in either optically clear bottom or standard microplates (*p* = 0.11 and *p* = 0.9, respectively, S1 Fig).

### Quantification of *C*. *trachomatis* IFUs via ICW

**Fig 1:**
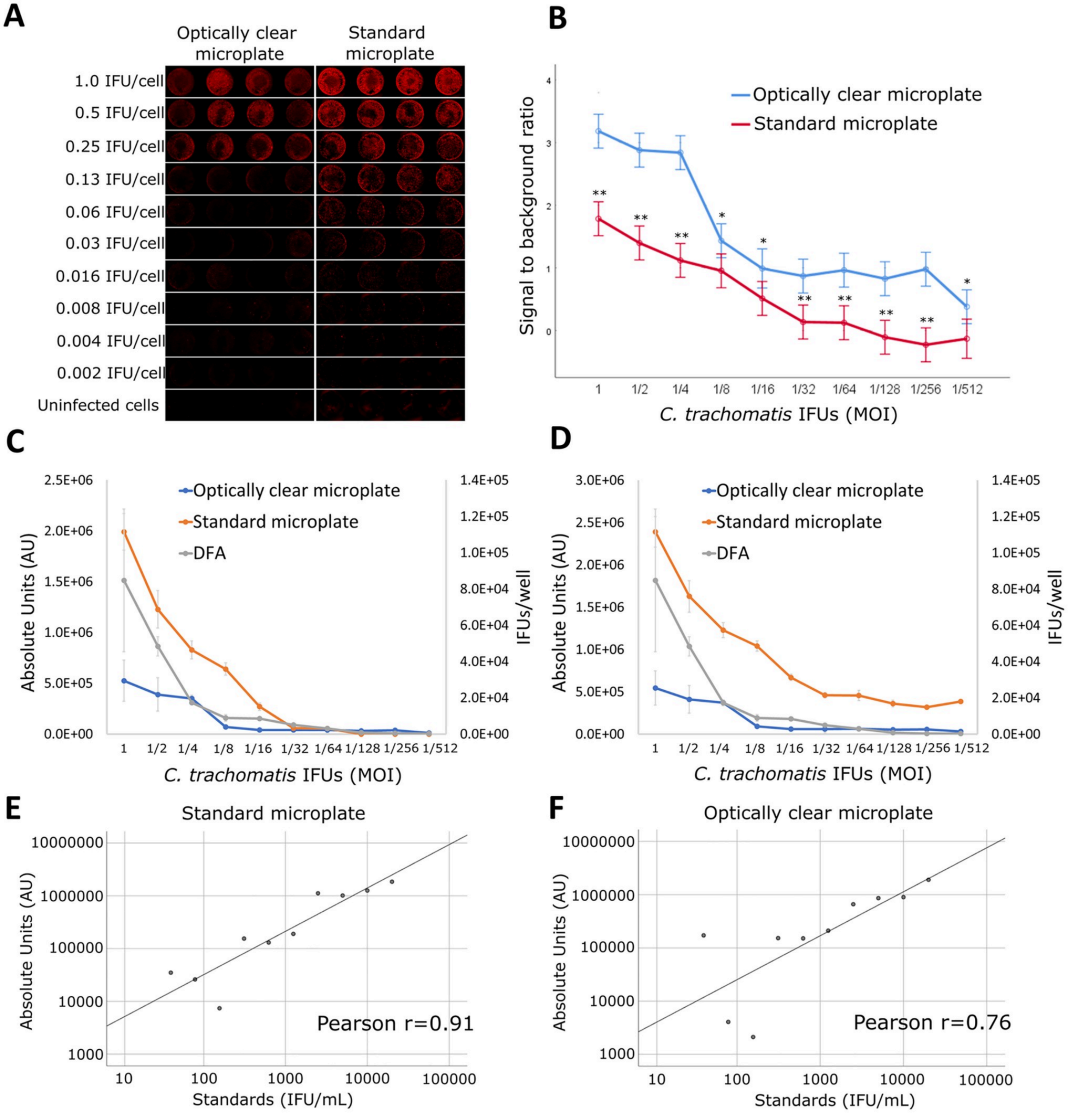
Standard curves of *C*. *trachomatis* IFUs via ICW assay in optically clear bottom and standard microplates, related to the enumeration of chlamydial IFUs by DFA assay. ICW assay: confluent McCoy cell monolayers, grown on either 96-well standard polystyrene or optically clear bottom cell culture microplates, were infected with two-fold serial dilutions of *C*. *trachomatis* EB suspension, from MOI of 1.0 to 1/2^9^ IFUs/cell. After 36 hours post infection, infected cell monolayers were fixed in 4% PFA, permeabilized by 0.1% triton x-100 in PBS, stained and scanned via Odyssey CLx as described in Materials and Methods. DFA: confluent McCoy cell monolayers, grown on coverslips in 24-well cell culture microplates, were infected as above described. After 36 hours post infection, infected cell monolayers were fixed in 96% ice cold methanol, stained and visualized via fluorescence microscopy (400X magnification) as described in Materials and Methods. (A) Representative infrared scan images of Chlamydia-infected cells on standard polystyrene cell-culture or optically clear bottom microplates from at least three independent experiments; (B) Signal to noise ratio of Chlamydia-infected cells in either standard polystyrene cell-culture or optically clear bottom microplates; Standard curves of *C*. *trachomatis* IFUs via ICW in optically clear bottom and standard microplates, calculated from near-infrared absorbance data with background (C) or no-background (D) subtraction, related to the enumeration of chlamydial IFU via DFA; Linear regression models of standard curves by the ICW assay after log-transformation on (E) standard polystyrene cell-culture microplates or (F) optically clear bottom microplates. **, *p* < 0.001; *, *p* < 0.05.

In order to evaluate the performance of ICW assay in the quantification of *C*. *trachomatis* via Odyssey CLx, we used as comparison the DFA assay on cell cultures, the traditional method employed for determining chlamydial titer. Specifically, we first set-up a standard curve of *C*. *trachomatis* IFU via ICW in relation to the enumeration of chlamydial inclusions determined via DFA (Fig 1). To this aim, cell monolayers were infected with known quantities of chlamydial EBs, from 2x10^5^ to 39 EBs/well (MOI from 1 to 1/2^9^, respectively), fixed, stained and scanned at 36h via Odyssey CLx (Fig 1A). Similar experiments were also performed on cell monolayers grown on 24-well plates and analysed via DFA. Subsequently, *C*. *trachomatis* titer (IFU/mL) was determined in different unknown chlamydial EB suspensions by using both methodologies. Lastly, the performance of ICW assay in the quantification of *C*. *trachomatis* was evaluated in two different microplates, standard and with optically clear well bottom.

According to general linear model with signal to noise ratio (log-transformed) as dependent variable, microplate type (optically clear vs. standard microplate) and dilution (from 1 to 1/2^9^) as between-units factors, both main effects [microplate type: F(1,58) = 253.88, p<0.001 and dilution: F(9,58) = 78.29, p<0.001] as well as microplate type*dilution interaction [F(9,58) = 5.37, p<0.001] resulted significant. These findings are represented in Fig 1B, where the signal to noise ratio of ICW assay in both microplates decreased with decreasing MOI, and it was generally higher in the optically clear bottom microplates as compared to the standard microplates. Specifically, signal-to-noise ratio was higher at each dilution level (Bonferroni adjusted p-values <0.05, consistently) even if differences between microplate type varied across dilution levels, as indicated by the significant interaction.

However, an increased sensitivity of ICW assay was not observed when optically clear bottom microplates were used. In fact, as shown in Fig 1C and 1D, the limits of detection, expressed as MOI, were 1/2^5^ and 1/2^4^, in the standard and optically clear bottom microplates, respectively; the ICW assay was also able to discriminate differences in MOI up to 1.0 in both microplates. After the appropriate log-transformation to make additive originally multiplicative variables, linear correlation between instrument readings and fixed standards was higher for standard microplates (r = 0.91, Fig 1E) than for optically clear bottom microplates (r = 0.76, Fig 1F). By contrast, DFA assay showed a sensitivity up to MOI 1/2^9^, as evidenced in Fig 1C and 1D.

**Fig 2:**
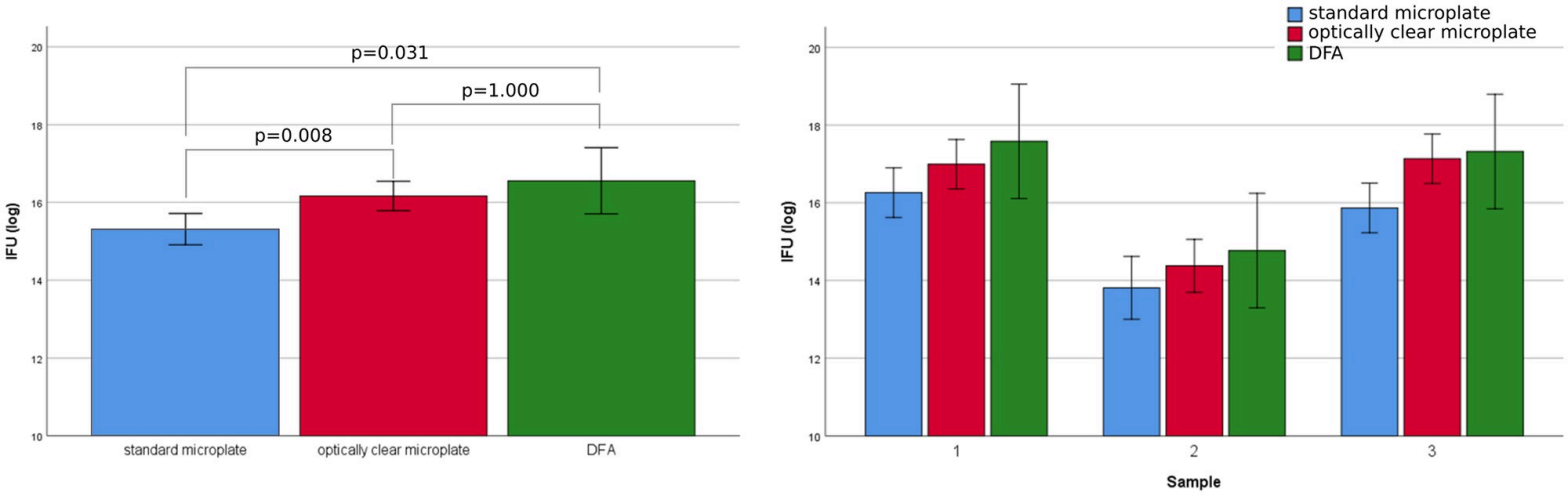
Quantification of *C*. *trachomatis* IFUs (IFU/mL) in samples with unknown chlamydial concentration via In-cell western and DFA assays. Bars represent mean values of log-transformed measures and error bars correspond to 95% confidence intervals (CI). The left panel shows the marginal means across the three samples and Bonferroni adjusted p-values for each of the three pairwise comparisons. The right panel shows the means (and 95% CI) for each sample (post-hoc comparisons are not reported since no evidence of Method*Sample interaction was found (p>0.80)).

When the number of chlamydial EB was estimated in the samples with unknown *C*. *trachomatis* concentrations, according to the relationship derived from the above regression models (ANOVA with log(ICW) as dependent variable and method (ICW in standard or optically clear microplates, and DFA) and samples (1, 2, 3) as between-measures factors), method resulted significant [F(2, 88) = 6.26; p = 0.003], without any evidence of dependence on samples [F(4, 88) = 0.31; p = 0.972]. As shown in Table 3 and in Fig 2, ICW measures (IFU/mL) were lower in standard microplates than in optically clear microplates (Bonferroni adjusted p = 0.008). ICW measures in standard microplates were also lower than DFA values (IFU/mL) (p = 0.031). On the other hand, no significant difference was observed between the ICW measures in optically clear microplates and DFA values (p = 1.000).

**Table 3:** Quantification of *C*. *trachomatis* IFUs (IFU/mL) in samples with unknown chlamydial concentration via In-cell western and DFA assays.

Samples	In-cell western assay						DFA		
	Optically clear microplate			Standard microplate					
**IFU/mL (log)**									
	**Mean**	**95% CI**		**Mean**	**95% CI**		**Mean**	**95% CI**	
**1**	16.261	15.623	16.899	16.991	16.353	17.629	17.581	16.108	19.055
**2**	13.81	13.003	14.617	14.377	13.695	15.059	14.769	13.296	16.242
**3**	15.868	15.23	16.505	17.134	16.496	17.772	17.318	15.844	18.791
**All**	15.313	14.909	15.716	16.168	15.791	16.545	16.556	15.706	17.407
**IFU/mL**									
	**Mean**	**95% CI**		**Mean**	**95% CI**		**Mean**	**95% CI**	
**1**	1.15E+07	6.10E+06	2.18E+07	2.39E+07	1.26E+07	4.53E+07	4.32E+07	9.90E+06	1.89E+08
**2**	9.95E+05	4.44E+05	2.23E+06	1.75E+06	8.86E+05	3.47E+06	2.59E+06	5.95E+05	1.13E+07
**3**	7.79E+06	4.11E+06	1.47E+07	2.76E+07	1.46E+07	5.23E+07	3.32E+07	7.60E+06	1.45E+08
**All**	4.47E+06	2.98E+06	6.69E+06	1.05E+07	7.21E+06	1.53E+07	1.55E+07	6.62E+06	3.63E+07

DFA, Direct immunofluorescence Assay; CI, Confidence Interval; IFU, Inclusion Forming Unit.

### *C*. *trachomatis* susceptibility to erythromycin via ICW assay

In order to evaluate the performance of ICW assay as a novel approach for screening anti-chlamydial drugs, erythromycin, one of the antibiotics recommended for treating *C*. *trachomatis* infections [26], was chosen and DFA assay was used as comparison. Specifically, the susceptibility of *C*. *trachomatis* to erythromycin was investigated via ICW and DFA assays by challenging *Chlamydia*-infected cell monolayers on either standard or optically clear bottom microplates with twofold serial dilutions of erythromycin (from 1 μg/mL to 1/2^9^ μg/mL).

**Fig 3:**
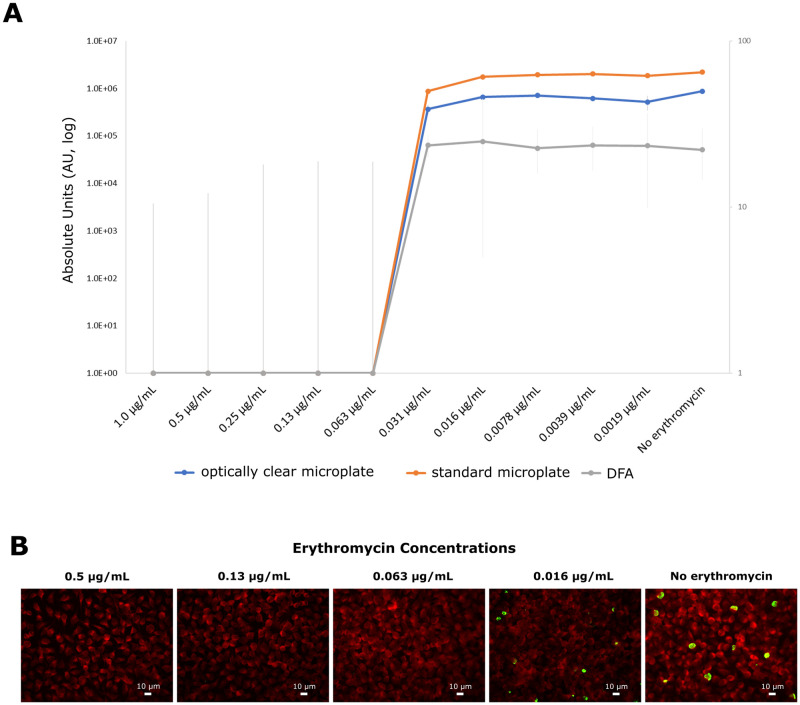
Susceptibility of *C*. *trachomatis* to erythromycin by ICW and DFA assays. Confluent McCoy cell monolayers, grown in either 96-well cell culture microplates or on coverslips in 24 well cell culture microplates, were infected with *C*. *trachomatis* EBs at a MOI of 1.0. Subsequently, cell monolayers were challenged with two-fold serial dilutions of erythromycin (from 1.0 μg/mL to 0.0019 μg/mL) and, then, incubated for 36 hours at 37°C and 5% CO_2_. 96-well microplates were then fixed in 4% PFA, permeabilized by 0.1% triton x-100 in PBS and analysed via Odyssey CLx, whereas 24-well microplates were fixed with 96% ice cold methanol and analysed via fluorescence microscopy. (A) Near-infrared absorbance data via ICW and chlamydial IFU via DFA in relation to erythromycin concentrations; (B) Immunohistological staining of infected cell monolayers treated with the erythromycin concentrations corresponding at MIC (0.063 μg/mL) and MIC_TP_ (0.0019 μg/mL) and untreated cells.

As evidenced in Fig 3, the ICW as well as the DFA assays were able to determine the MIC; via DFA assay, the MIC_TP_ was 1/2^5^ and, hence, the MIC value was 1/2^4^ μg/mL. The ICW assay detected the same MIC value as the DFA assay, at 1/2^4^ μg/mL. Furthermore, no differences in the MIC value were observed when the ICW assay was used on either standard or optically clear-bottom microplates.

## Discussion

The findings of our study show that the ICW assay can represent a simple, affordable, and fast alternative to DFA assay on cell-cultures for either the quantification of chlamydial inclusions or the susceptibility testing to anti-chlamydial agents. In particular, the ICW assay possesses high accuracy, as evidenced by the low dispersion of the standard curve for chlamydial quantification observed in our study. Furthermore, a very high concordance between ICW and DFA assay was observed in the enumeration of chlamydial IFUs as well as the determination of erythromycin MIC. Also, ICW assay significantly reduce the analysis duration (approximately 3 hours) as compared to the DFA assay, that requires from one to several days in relation to the number of samples analysed. Lastly, the performance of the ICW assay is not significantly influenced by the type of microplate used. This is an important aspect for reducing the overall cost of the analysis, since the optically clear bottom microplates are particularly expensive.

Historically, the well-established approaches, such as DFA on cell-cultures, have been adopted for the quantification of *C*. *trachomatis* EBs and the testing of chlamydial susceptibility to antibiotics [16, 19, 20]. However, DFA assay rely on investigator’s experience in identifying chlamydial EBs, and the number of samples assayed per day is limited, making it not very time-efficient [20]. As a consequence, the DFA on cell cultures is not routinely employed in all laboratories and require dedicated facilities and trained professionals.

Recently, other alternative approaches have been suggested for improving or automating *C*. *trachomatis* quantification or susceptibility testing. In particular, genetically modified *C*. *trachomatis* strains expressing Green Fluorescent Protein (GFP), *in-silico* analysis of ImmunoSpot data or modified plaque-forming assays [27–31] have been proposed. However, these approaches have not gained a lot of traction in the scientific community since, for example, the engineering of GFP-producing *C*. *trachomatis* is challenging and time-consuming [27–31], the ImmunoSpot imaging system shows a low resolving power, underestimating chlamydial quantification [30], and the plaque assay lack accuracy and reproducibility [31].

Differently, the ICW assay possesses numerous advantages. First, ICW assay removes the investigator’s bias associated with the subjective microscopic examination of DFA assay, increasing, thus, the accuracy of results. Second, the ICW assay shows the potential to greatly increase the throughput of the analysis for its ability to scan up to 384-well microplates, allowing the simultaneous testing of several anti-chlamydial agents. Lastly, the ICW possesses further advantages as compared to the other methodologies based on the same test principle, like the horseradish peroxidase (HRP) based ELISA assay. The ICW assay, indeed, improve the reliability of *C*. *trachomatis* inclusion enumeration via the quantification of cell monolayer integrity, since it is able to acquire two different signals simultaneously by using fluorescent molecules emitting at different wavelength, differently from the HRP-ELISA. Also, the ICW assay can be stored at -20°C up to a month, making it possible to revisit the results of the assay at a later time, whereas HRP-ELISA must be read in the first 15 minutes after the addition of the stop solution. Nevertheless, HRP-ELISA is a well-known methodology in clinical laboratories and has been used for antimicrobial susceptibility testing towards non-bacterial pathogens [32, 33].

Overall, the ICW assay may be a promising candidate as an accurate and accessible methodology for *C*. *trachomatis* antimicrobial susceptibility testing, since it can be completely automated and, hence, within reach of all laboratories.

## Supporting information

S1 Fig Stability of IRdye 680RD after 1 month of storage at -20°C. Absorbance values (AU) in relation to C. *trachomatis* MOI determined by Odyssey CLx in optically clear bottom and standard microplates scanned at day O and after 1 month of storage at -20°C.
(TIF)

S1 File (XLSX)

## References

[1] Fenwick A. The global burden of neglected tropical diseases. Public Health. 2012;126(3):233–236. 10.1016/j.puhe.2011.11.015 22325616

[2] Report on global sexually transmitted infection surveillance, 2018. Geneva: World Health Organization; 2018. Licence: CC BY-NC-SA 3.0 IGO.

[3] Di Pietro M, Filardo S, Romano S, Sessa R. *Chlamydia trachomatis* and *Chlamydia pneumoniae* Interaction with the Host: Latest Advances and Future Prospective. Microorganisms. 2019;7(5):140. 10.3390/microorganisms7050140 31100923 PMC6560445

[4] Filardo S, Skilton RJ, O’Neill CE, Di Pietro M, Sessa R, Clarke IN. Growth kinetics of *Chlamydia trachomatis* in primary human Sertoli cells. Sci Rep. 2019;9(1):5847. 10.1038/s41598-019-42396-3 30971744 PMC6458130

[5] O’Connell CM, Ferone ME. *Chlamydia trachomatis* Genital Infections. Microb Cell. 2016;3(9):390–403. 10.15698/mic2016.09.525 28357377 PMC5354567

[6] Filardo S, Di Pietro M, Tranquilli G, Latino MA, Recine N, Porpora MG, et al. Selected Immunological Mediators and Cervical Microbial Signatures in Women with *Chlamydia trachomatis* Infection. mSystems. 2019;4(4):e00094–19. 10.1128/mSystems.00094-19 31164450 PMC6550367

[7] Chiarelli TJ, Grieshaber NA, Omsland A, Remien CH, Grieshaber SS. Single-Inclusion Kinetics of *Chlamydia trachomatis* Development. mSystems. 2020;5(5):e00689–20. 10.1128/mSystems.00689-20 33051378 PMC7567582

[8] Sessa R, Di Pietro M, Filardo S, Bressan A, Mastromarino P, Biasucci AV, et al. Lactobacilli-lactoferrin interplay in *Chlamydia trachomatis* infection. Pathog Dis. 2017;75(5). 10.1093/femspd/ftx054 28505248

[9] Soules KR, LaBrie SD, May BH, Hefty PS. Sigma 54-Regulated Transcription Is Associated with Membrane Reorganization and Type III Secretion Effectors during Conversion to Infectious Forms of *Chlamydia trachomatis*. mBio. 2020;11(5):e01725–20. 10.1128/mBio.01725-20 32900805 PMC7482065

[10] Skilton RJ, Cutcliffen LT, Barlow D, Wang Y, Salim O, Lambden PR, et al. Penicillin induced persistence in Chlamydia trachomatis: high quality time lapse video analysis of the developmental cycle. PLoS One. 2009;4(11):e7723. 10.1371/journal.pone.0007723 19893744 PMC2769264

[11] Shao L, You C, Cao J, Jiang Y, Liu Y, Liu Q. High treatment failure rate is better explained by resistance gene detection than by minimum inhibitory concentration in patients with urogenital *Chlamydia trachomatis* infection. Int J Infect Dis. 2020;96:121–127. 10.1016/j.ijid.2020.03.015 32173573

[12] Mohammadzadeh F, Dolatian M, Jorjani M, Afrakhteh M, Majd HA, Abdi F, et al. Urogenital *Chlamydia trachomatis* treatment failure with azithromycin: A meta-analysis. Int J Reprod Biomed. 2019;17(9):603–620. 10.18502/ijrm.v17i9.5093 31646255 PMC6804326

[13] Janssen KJ, Hoebe CJ, Dukers-Muijrers NH, Eppings L, Lucchesi M, Wolffs PF. Viability-PCR Shows That NAAT Detects a High Proportion of DNA from Non-Viable *Chlamydia trachomatis*. PLoS One. 2016;11(11):e0165920. 10.1371/journal.pone.0165920 27812208 PMC5094775

[14] Phillips S, Vodstrcil LA, Huston WM, Lawerence A, Timms P, Chen MY, et al. Detection of *Chlamydia trachomatis* mRNA using digital PCR as a more accurate marker of viable organism. Eur J Clin Microbiol Infect Dis. 2018;37(11):2117–2122. 10.1007/s10096-018-3347-y 30109584

[15] Shao L, Guo Y, Jiang Y, Liu Y, Wang M, You C, et al. Sensitivity of the Standard *Chlamydia trachomatis* Culture Method Is Improved After One Additional In Vitro Passage. J Clin Lab Anal. 2016;30(5):697–701. 10.1002/jcla.21924 26987564 PMC6807024

[16] Mahmud NU, Hossain MA, Nahar K, Ahmed GS, Mahmud C, Paul SK, et al. Non-culture diagnosis of *Chlamydia trachomatis* genital infection in sexually active women. Mymensingh Med J. 2012;21(1):8–12. 22314447

[17] Pitt R, Alexander S, Ison C, Horner P, Hathorn E, Goold P, et al. Phenotypic antimicrobial susceptibility testing of *Chlamydia trachomatis* isolates from patients with persistent or successfully treated infections. J Antimicrob Chemother. 2018;73(3):680–686. 10.1093/jac/dkx454 29207004

[18] Eszik I, Lantos I, Önder K, Somogyvári F, Burián K, Endrész V, et al. High dynamic range detection of *Chlamydia trachomatis* growth by direct quantitative PCR of the infected cells. J Microbiol Methods. 2016;120:15–22. 10.1016/j.mimet.2015.11.010 26578244

[19] Suchland RJ, Geisler WM, Stamm WE. Methodologies and cell lines used for antimicrobial susceptibility testing of *Chlamydia* spp. Antimicrob Agents Chemother. 2003;47(2):636–42. 10.1128/aac.47.2.636-642.2003 12543671 PMC151736

[20] Jespersen DJ, Flatten KS, Jones MF, Smith TF. Prospective comparison of cell cultures and nucleic acid amplification tests for laboratory diagnosis of Chlamydia trachomatis Infections. J Clin Microbiol. 2005;43(10):5324–6. 10.1128/JCM.43.10.5324-5326.2005 16208009 PMC1248517

[21] Janssen KJH, Dirks JAMC, Dukers-Muijrers NHTM, Hoebe CJPA, Wolffs PFG. Review of Chlamydia trachomatis viability methods: assessing the clinical diagnostic impact of NAAT positive results. Expert Rev Mol Diagn. 2018;18(8):739–747. 10.1080/14737159.2018.1498785 29987959

[22] Sarshar M, Scribano D, Tranquilli G, Di Pietro M, Filardo S, Zagaglia C, et al. A simple, fast and reliable scan-based technique as a novel approach to quantify intracellular bacteria. BMC Microbiol. 2019 11 12;19(1):252. 10.1186/s12866-019-1625-1 31718545 PMC6849193

[23] Boveia V, Schutz-Geschwender A. Quantitative Analysis of Signal Transduction with In-Cell Western Immunofluorescence Assays. Methods Mol Biol. 2015;1314:115–30. 10.1007/978-1-4939-2718-0_13 26139260

[24] Skilton RJ, Wang Y, O’Neill C, Filardo S, Marsh P, Bénard A, et al. The *Chlamydia muridarum* plasmid revisited: new insights into growth kinetics. Wellcome Open Res. 2018;3:25. 10.12688/wellcomeopenres.13905.1 29657985 PMC5871946

[25] Sessa R, Di Pietro M, De Santis F, Filardo S, Ragno R, Angiolella L. Effects of Mentha suaveolens essential oil on *Chlamydia trachomatis*. Biomed Res Int. 2015;2015:508071. 10.1155/2015/508071 25685793 PMC4320923

[26] World Health Organization. WHO guidelines for the treatment of Chlamydia trachomatis. World Health Organization. 2016. 27559553

[27] Zhang Y, Xian Y, Gao L, Elaasar H, Wang Y, Tauhid L, et al. Novel Detection Strategy To Rapidly Evaluate the Efficacy of Antichlamydial Agents. Antimicrob Agents Chemother. 2017;61(2):e02202–16. 10.1128/AAC.02202-16 27855081 PMC5278686

[28] Zuck M, Feng C, Hybiske K. Using Fluorescent Proteins to Visualize and Quantitate *Chlamydia* Vacuole Growth Dynamics in Living Cells. J Vis Exp. 2015;(104):51131. 10.3791/51131 26484535 PMC4692645

[29] Vromman F, Laverrière M, Perrinet S, Dufour A, Subtil A. Quantitative monitoring of the *Chlamydia trachomatis* developmental cycle using GFP-expressing bacteria, microscopy and flow cytometry. PLoS One. 2014;9(6):e99197. 10.1371/journal.pone.0099197 24911516 PMC4049595

[30] Wang S, Indrawati L, Wooters M, Caro-Aguilar I, Field J, Kaufhold R, et al. A novel automated method for enumeration of Chlamydia trachomatis inclusion forming units. J Immunol Methods. 2007;324(1–2):84–91. 10.1016/j.jim.2007.05.004 17553519

[31] Banhart S, Saied EM, Martini A, Koch S, Aeberhard L, Madela K, et al. Improved plaque assay identifies a novel anti-*Chlamydia* ceramide derivative with altered intracellular localization. Antimicrob Agents Chemother. 2014;58(9):5537–46. 10.1128/AAC.03457-14 25001308 PMC4135853

[32] Spurgers KB, Hurt CR, Cohen JW, Eccelston LT, Lind CM, Lingappa VR, et al. Validation of a cell-based ELISA as a screening tool identifying anti-alphavirus small-molecule inhibitors. J Virol Methods. 2013;193(1):226–31. 10.1016/j.jviromet.2013.06.007 23764417

[33] Conzelmann C, Gilg A, Groß R, Schütz D, Preising N, Ständker L, et al. An enzyme-based immunodetection assay to quantify SARS-CoV-2 infection. Antiviral Res. 2020;181:104882. 10.1016/j.antiviral.2020.104882 32738255 PMC7388004

