# Supplementary figures and images for: In-cell western assay as a high-throughput approach for Chlamydia trachomatis quantification and susceptibility testing to antimicrobials
Source: PLoS One. 2021 May 11;16(5):e0251075. doi: 10.1371/journal.pone.0251075 (PMC8112659; doi:10.1371/journal.pone.0251075)

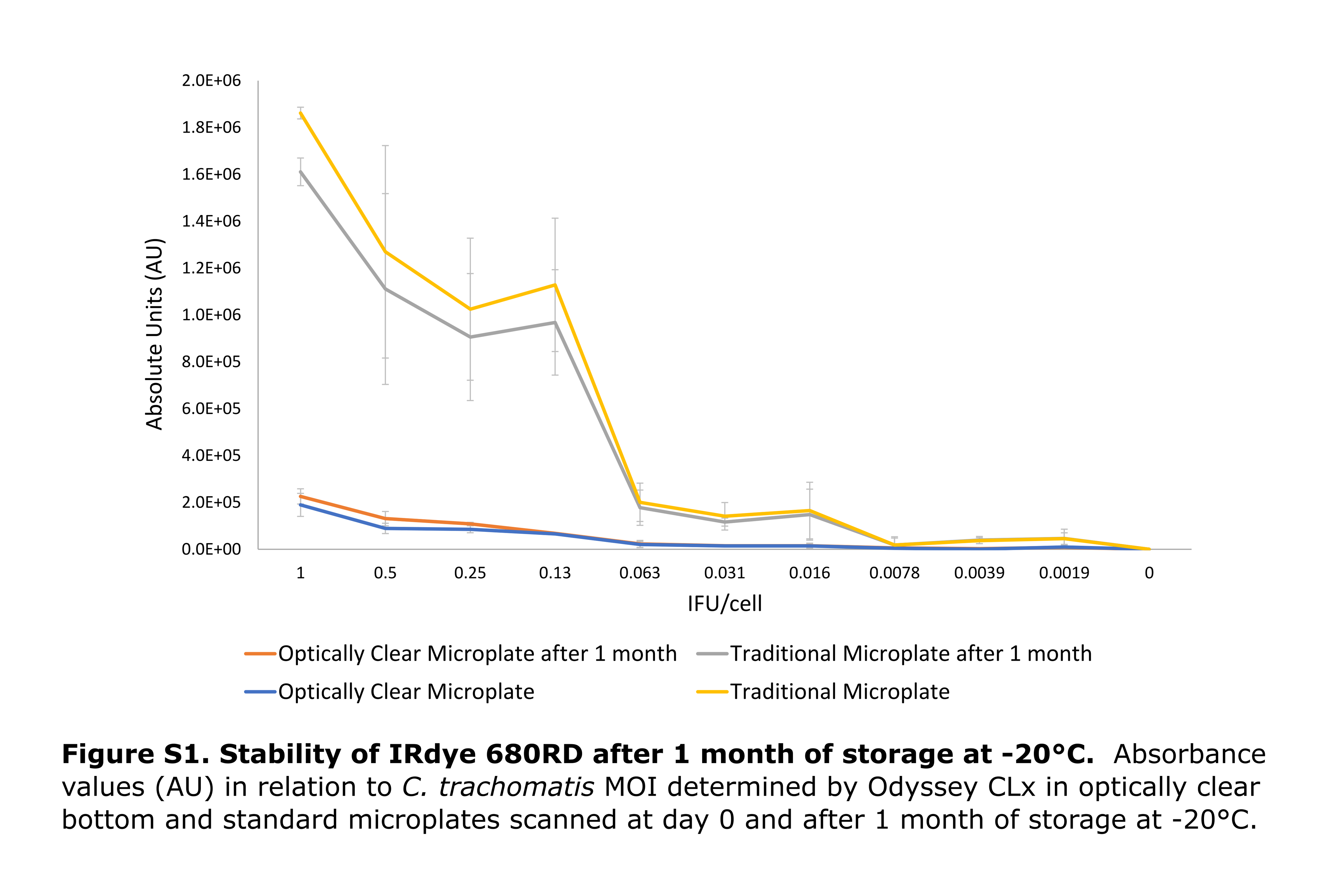

Supplement: S1 Fig — Absorbance values (AU) in relation to C. trachomatis MOI determined by Odyssey CLx in optically clear bottom and standard microplates scanned at day O and after 1 month of storage at -20°C. (TIF) [file pone.0251075.s001.tif]
